# Point-of-care ultrasound in primary care: a systematic review of generalist performed point-of-care ultrasound in unselected populations

**DOI:** 10.1186/s13089-019-0145-4

**Published:** 2019-11-19

**Authors:** Bjarte Sorensen, Steinar Hunskaar

**Affiliations:** 1 Hjelmeland General Practice Surgery, Prestagarden 13, 4130 Hjelmeland, Norway; 2 Department of Global Public Health and Primary Care, University of Bergen, Bergen, Norway; 3 National Centre for Emergency Primary Health Care, NORCE Norwegian Research Centre AS, Bergen, Norway

**Keywords:** General practice, Family medicine, Emergency medicine, Point-of-care ultrasound, Ultrasound

## Abstract

**Background:**

Both the interest and actual extent of use of point-of-care ultrasound, PoCUS, among general practitioners or family physicians are increasing and training is also increasingly implemented in residency programs. However, the amount of research within the field is still rather limited compared to what is seen within other specialties in which it has become more established, such as in the specialty of emergency medicine. An assumption is made that what is relevant for emergency medicine physicians and their populations is also relevant to the general practitioner, as both groups are generalists working in unselected populations. This systematic review aims to examine the extent of use and to identify clinical studies on the use of PoCUS by either general practitioners or emergency physicians on indications that are relevant for the former, both in their daily practice and in out-of-hours services.

**Methods:**

Systematic searches were done in PubMed/MEDLINE using terms related to general practice, emergency medicine, and ultrasound.

**Results:**

On the extent of use, we identified 19 articles, as well as 26 meta-analyses and 168 primary studies on the clinical use of PoCUS. We found variable, but generally low, use among general practitioners, while it seems to be thoroughly established in emergency medicine in North America, and increasingly also in the rest of the world. In terms of clinical studies, most were on diagnostic accuracy, and most organ systems were studied; the heart, lungs/thorax, vessels, abdominal and pelvic organs, obstetric ultrasound, the eye, soft tissue, and the musculoskeletal system. The studies found in general either high sensitivity or high specificity for the particular test studied, and in some cases high total accuracy and superiority to other established diagnostic imaging modalities. PoCUS also showed faster time to diagnosis and change in management in some studies.

**Conclusion:**

Our review shows that generalists can, given a certain level of pre-test probability, safely use PoCUS in a wide range of clinical settings to aid diagnosis and better the care of their patients.

## Background

Point-of-care ultrasound, PoCUS, can be defined as the use of an image-producing ultrasound device for diagnostic and procedural guidance, by the clinician himself, at the point of care, in real time allowing for direct correlation with signs and symptoms [1]. It is integrated in the clinical work, and may increase accuracy of diagnoses or aid procedures, as well as reduce time spent to diagnoses and decreased overall costs [2].

General practitioners (GPs), or family physicians, work in a range of settings and levels of urgencies, from daytime run clinics, through out-of-hours (OOH) services such as primary care urgent care centres, to the provision of undifferentiated emergency medicine in rural and remote regions. Globally, there are many different organisational models for OOH services, often running in parallel, including GP rota groups, cooperatives, primary care centres, as well as in-hospital emergency departments [3].

General practitioners are trained to manage both chronic conditions as well as acute emergencies, often within the same session, seeing women and men, young and old. In many countries, such as Australia [4] and Canada [5], general practitioners in rural and remote areas are expected to handle all emergencies and are often the only physicians available for initial diagnosis, management, and stabilisation within several hours of travel by road, water, or air. In countries such as Norway [6] and New Zealand [7], GPs are organised as part of the emergency response chain acting as a first responder and a team member to the ambulance services. Skills such as obtaining peripheral venous access and diagnosing life-threatening medical and traumatological conditions are expected [8, 9].

There are, therefore, many settings where the GP could potentially benefit from her own use of PoCUS. Both the interest and actual extent of use among GPs are increasing and PoCUS training is also increasingly implemented in residency programs [10]. However, the amount of research on PoCUS performed by GPs is still rather limited compared to other specialties in which it has become more established, such as in the specialty of emergency medicine [11, 12].

A recently published systematic review of PoCUS in general practice, identifying articles where the operators were GPs, concluded that it has the potential to be an important tool for the GP and possibly reduce health costs, but emphasises the need for further research [12]. Meanwhile, we think that it may be useful to also review studies where the setting is similar and the PoCUS operators also are, like GPs, physicians with a generalist training and perspective. We made the assumption that findings from studies where the operator is an emergency physician (EP) working in an unselected emergency department population also will be relevant for GPs.

The aim of this systematic review is thus twofold: first, to examine the extent of use among both GPs and EPs; second, to identify primary clinical research articles or meta-analyses on PoCUS for indications relevant for GPs in which the population is unselected (open GP practice or emergency departments) and the operators are either GPs or EPs.

## Methods

Systematic searches were performed in the PubMed databases. Indexed MEDLINE-articles were identified by medical subject headings’ (MeSH) keywords describing ultrasound, general practice, and emergency medicine (Table 1). Non-indexed PubMed articles were identified by corresponding keywords (Appendix 2 shows the exact search algorithm). The reference lists of included articles were also reviewed.

**Table 1:** Search algorithms

MeSH terms	Ultrasonography (included echocardiography)	Primary health care General practice (included family practice) General practitioners Physicians, primary care Physicians, family	Emergency medical services (included emergency service, hospital) Emergency medicine Emergency treatment Emergencies
Additional keywords used for search in non-indexed articles	Ultrasound POCUS Echocardiography	General practitioner Primary care physician Family physician	Emergency physician Prehospital medicine

Only studies involving the clinical use of two-dimensional image-producing ultrasound at the point of care were included. Studies on hospitalised inpatients were excluded, as well as studies where the operator was a non-generalist, non-physician, or prehospital emergency medical service personnel. Case studies or case series were excluded, as were the use of ultrasound on hyperacute indications or for procedures less likely to be of relevance to most general practitioners (Appendix 1). Meta-analyses where the majority of the included articles fit our inclusion criteria were included, and the individual studies analyzed by these meta-analyses were excluded from our review to avoid double treatment. Articles published after the latest meta-analyses were included, as were articles outside the scope of the meta-analyses identified. Articles in other languages than English, German, Spanish, or any of the Scandinavian languages were excluded. The search was last performed on 1 June 2019.

## Results

**Fig. 1:**
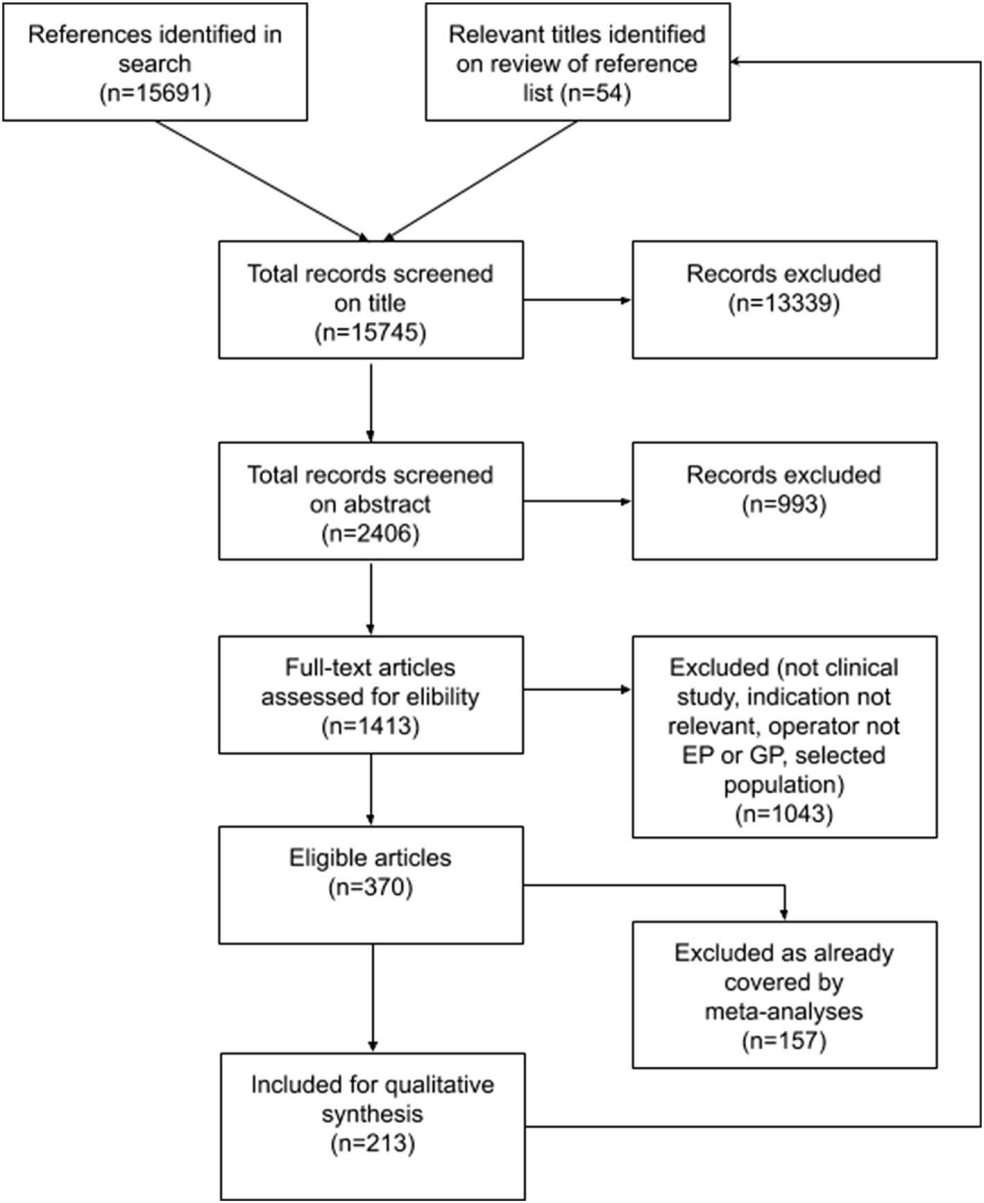
Study selection flow diagram

We identified 15,745 articles which were screened for eligibility, and after screening, 1413 full text articles we were left with 213 articles for inclusion, as shown in Fig. 1. Out of these, 19 were articles about the extent of use, while 26 were meta-analyses, and 168 primary research studies on PoCUS.

### The extent of use

There is great variation in the extent of use of PoCUS among GPs in Europe. In Norway, 23% of emergency primary care centres had access to their own ultrasound machines in 2015. However, only 1 of 15 of the GPs working there used ultrasound ever and only 0.3% of billings included an ultrasound item [13]. Ultrasound was in 2014 commonly used in Germany (about 45%) and Greenland (about two-thirds), while it was less commonly used in Sweden, Denmark, Austria, and Catalonia (< 1%) [14]. GPs, and EPs, working in emergency departments in rural Canada had good access to ultrasound equipment already in 2013 and increasingly until today (60–95%), while between 44 and 76% reported, they used ultrasound, a third of these on every shift [15–17].

Among EPs, ultrasound was used in 5% of the consultations in emergency departments in France in 2014 [18]. French emergency departments (EDs) have seen an increase in the availability of ultrasound equipment from 52 to 71% between 2011 and 2016 [19]. EPs had access to ultrasound equipment in 89% of Danish emergency departments in 2013 [20]. In China, 54% of EPs reported having access to equipment in 2016, and 43% of respondents reported using PoCUS in their clinical work [21]. In South Korea, it was available in 2014 in all surveyed EDs and 82.7% of respondents used PoCUS daily on adult patients, but only 23.6% performed paediatric PoCUS daily [22]. In Colombia, 57% of all emergency medicine residents responded that they lacked equipment, while 52% responded that they had used ultrasound during their training [23]. The use of PoCUS is integrated in the emergency physician training in the USA [24], and from 2004 to 2015, the access to equipment in emergency departments has risen from 19% to between 66 and 96%, and the lack of physician training is now seen as the major barrier rather than the lack of available technology [25–30].

### Relevant indications

We found 26 meta-analyses and 168 primary studies on PoCUS used by generalists on a wide range of indications that we deemed relevant for the general practitioner, and they have been sorted according to the relevant organ systems: heart, lungs, vessels, abdomen, obstetric ultrasound, the eye; soft tissue, and musculoskeletal system.

The most studied parameter was diagnostic accuracy, and Tables 2, 3, 4, 5, 6, 7 and 8 show the test characteristics of a multitude of examinations. The sensitivities and specificities are displayed, and 95% confidence intervals are included where available. Positive and negative likelihood ratios (LR+/LR−) have been listed rather than positive and negative predictive values, as the former are prevalence independent, while the latter is only valid for the given prevalence in the studied population. Where either of the tabulated parameters was not available, we calculated these from the given data and indicated as such in the tables. Where available, the amount of time spent on specific didactic teaching is listed.

**Table 2:** Summary of test accuracy findings in echocardiography

Test	Author	Op.	Year	Country or MA (studies)	Train.	*n*	Prev (%)	Age (years)	Criterion standard	Sn. (%) (95% CI)	Sp. (%) (95% CI)	LR+ (95% CI)	LR− (95% CI)
MAPSE < 10 mm	Mjølstad et al. [33]	GP	2012	Norway	8 h.	92	NR	73	Cardiologist echo.	83.3 (66.4–92.7)	77.6 (64.1–87.1)	3.72^a^	0.746^a^
LVH (ventricular wall > 13 mm)	Evangelista et al. [34]	GP	2013	Spain	NR	393	46	71	Cardiologist echo.	89.8 (NR)	98.4 (NR)	56.1^a^	0.114^a^
	Evangelista et al. [35]	GP	2016	Spain	NR	1312	16	67	Cardiologist echo.	71.4 (63.1–79.7)	97.4 (96.7–98.6)	27.5^a^	0.29^a^
LVEF < 50–55%	Unlüer et al. [41]	EP	2014	Turkey	NR	133	56	70	Cardiologist echo.	98.7 (91.8–99.9)	86.2 (74.1–93.4)	7.15 (3.76–13.6)	0.015 (0.002–0.109)
	*Martindale et al. [*55*]*	*EP*	*2016*	*MA* (*3*)	*NR*	*325*	*41*	*NR*	*Final diagnosis*	*80.6* (*72.9*–*86.9*)	*80.6* (*74.3*–*86.0*)	*4.1* (*2.4*–*7.2*)	*0.24* (*0.17*–*0.35*)
	Shah et al. [42]	EP	2016	Haiti	30 h.	117	40	36	Cardiologist echo.	93.6 (81.4–98.3)	100 (93.5–100)	∞^a^	0.064^a^
	Farsi et al. [43]	EP	2017	Iran	10 h.	205	51	61	Cardiologist echo.	89 (81–99)	96 (90–99)	22 (8–58)	0.12 (0.07–0.20)
LVEF < 40%	Dehbozorgi et al. [44]	EP	2019	Iran	NR	100	28	58	Final diagnosis (AHF)	100 (88–100)	88 (78–94)	8 (4.34–14.74)	0
LV dysfunction	Evangelista et al. [35]	GP	2016	Spain	NR	1312	4	67	Cardiologist echo.	50.0 (30.4–69.6)	92.7 (91.3–94.2)	6.85^a^	0.539^a^
LA dilatation	Evangelista et al. [35]	GP	2016	Spain	NR	1312	4	67	Cardiologist echo.	41.5 (25.2–57.8)	97.7 (96.8–98.6)	18.0^a^	0.701^a^
RVD	Evangelista et al. [34]	GP	2013	Spain	NR	393	22	71	Cardiologist echo.	80.2 (NR)	98.9 (NR)	73.9^a^	0.200^a^
	Farsi et al. [43]	EP	2017	Iran	10 h.	205	16	61	Cardiologist echo.	98 (94–99)	87 (69–96)	41 (15–109)	0.07 (0.02–0.27)
RVP	Farsi et al. [43]	EP	2017	Iran	10 h.	205	3	61	Cardiologist echo.	100 (52–100)	100 (98–100)	∞^a^	0^a^
Aortic valve sclerosis	Evangelista et al. [34]	GP	2013	Spain	NR	393	23	71	Cardiologist echo.	81.6 (NR)	98.2 (NR)	45.3^a^	0.187^a^
Aortic stenosis	Evangelista et al. [35]	GP	2016	Spain	NR	1312	5	67	Cardiologist echo.	50.0 (36.1–64.0)	98.1 (97.0–99.1)	26.3^a^	0.510^a^
Aortic insufficiency	Evangelista et al. [34]	GP	2013	Spain	NR	393	27	71	Cardiologist echo.	86.1 (NR)	95.7 (NR)	76.9^a^	0.145^a^
	Evangelista et al. [35]	GP	2016	Spain	NR	1312	4	67	Cardiologist echo.	58.3 (43.3–73.3)	99.0 (98.3–99.6)	58.3^a^	0.421^a^
Dilated ascending aorta	Evangelista et al. [34]	GP	2013	Spain	NR	393	15	71	Cardiologist echo.	89.1 (NR)	100 (NR)	∞^a^	0.109^a^
	Evangelista et al. [35]	GP	2016	Spain	NR	1312	9	67	Cardiologist echo.	54.1 (37.1–70.2)	99.1 (98.4–99.6)	60.1^a^	0.463^a^
Mitral insufficiency	Evangelista et al. [34]	GP	2013	Spain	NR	393	48	71	Cardiologist echo.	89.1 (NR)	87.2 (NR)	6.96^a^	0.125^a^
	Evangelista et al. [35]	GP	2016	Spain	NR	1312	6	67	Cardiologist echo.	72.7 (61.2–84.2)	97.7 (96.8–98.6)	31.6^a^	0.279^a^
Mitral stenosis	Evangelista et al. [35]	GP	2016	Spain	NR	1312	1	67	Cardiologist echo.	62.8 (22.7–100)	98.1 (97.3–98.9)	33.1^a^	0.379^a^
Tricuspid insufficiency	Evangelista et al. [35]	GP	2016	Spain	NR	1312	4	67	Cardiologist echo.	41.4 (21.7–61.0)	98.9 (98.3–99.5)	37.6^a^	0.694^a^
Hypertrophic cardiomyopathy	Evangelista et al. [35]	GP	2016	Spain	NR	1312	1	67	Cardiologist echo.	44.4 (6.4–82.5)	99.8 (99.6–100)	222^a^	0.557^a^
Diastolic heart failure	Unlüer et al. [47]	EP	2012	Turkey	6 h.	69	74	63	Cardiologist echo.	89 (77–95)	80 (51–95)	4.5 (1.6–12)	0.14 (0.06–0.21)
	Ehrman et al. [48]	EP	2015	USA	3 h.	62	52	56	Cardiologist echo.	92 (60–100)	69 (50–83)	2.9	0.12
Restrictive mitral pattern	Nazerian et al. [50]	EP	2010	Italy	4 h.	125	35	78	Final diagnosis of AHF	82 (73–87)	90 (84–94)	8.27 (4.57–15.42)	0.21 (0.14–0.32)
Wall motion abnormality	Farsi et al. [43]	EP	2017	Iran	10 t.	205	33	61	Cardiologist echo.	97 (89–99)	87% (80–92)	8 (5–12)	0.03 (0.01–0.13)
	Croft et al. [52]	EP	2019	USA	NR	75	62	65	Cardiologist echo. or ventriculogram	88 (75–96)	92 (75–99)	11.5 (3.1–43.7)	0.13 (0.05–0.29)
Speckle tracking	Reardon et al. [53]	EP	2018	USA	NR	75	16	52	Cardiologist echo. or final diagnosis ACS	29 (17–46)	88 (72–96)	2.4^a^	0.81^a^
Pericardial effusion	Mandavia et al. [54]	EP	2001	USA	5 h.	515	20	NR	Cardiologist echo.	96.0 (90.4–98.9)	98.0 (95.8 to 99.1)	48.0^a^	0.0408^a^
	Farsi et al. [43]	EP	2017	Iran	10 h.	205	10	61	Cardiologist echo.	86 (63–96)	96 (91–98)	20 (10–40)	0.15 (0.05–0.40)
	Shah et al. [42]	EP	2016	Haiti	30 h.	117	8	36	Cardiologist echo.	88.9 (50.7–99.4)	99.1 (94.2–100)	98.8^a^	0.112^a^
	Bustam et al. [39]	EP	2014	Malaysia	3 h.	100	5	NR	Cardiologist echo.	60 (15^a^–95^a^)	100 (96^a^–100^a^)	∞^a^	0.40^a^

MA, meta-analysis (shown in italics with number of studies in brackets); Op., operators; Train., time spent in didactic intervention; *n*, size of population; Prev., prevalence; Age, median or mean age in years; Sn., sensitivity; Sp., specificity; CI, confidence interval; LR+, positive likelihood ratio; LR−, negative likelihood ratio; NR, not reported; echo., echocardiography; MAPSE, mitral annular plane systolic excursion; GP, general practitioner; LVH, left-ventricular hypertrophy; LVEF, left-ventricular ejection fraction; EP, emergency physician; LV, left ventricle; LA, left atrial; RVD, right-ventricular dilatation; RVP, right-ventricular pressure

^a^Calculated by the authors

**Table 3:** Summary of test accuracy findings in lung ultrasound

Test	Author	Op.	Year	Country (no. of studies in MA)	Train.	*n*	Prev (%)	Age	Criterion standard	Sn. in % (95% CI)	Sp. in % (95% CI)	LR+ (95% CI)	LR− (95% CI)
Diffuse interstitial syndrome in heart failure	*Martindale et al. [*55*]*	*EP*	*2016*	*MA* (*8*)	*NR*	*1914*	*48*	*NR*	*Final diagnosis*	*85.3* (*82.8*–*87.5*)	*92.7* (*90.9*–*94.3*)	*7.4* (*4.2*–*12.8*)	*0.16* (*0.05*–*0.51*)
	*McGivery et al. [*56*]*	*EP*	*2018*	*MA* (*5*)^c^	*NR*	*1387*	*NR*	*NR*	*Final diagnosis*	*88.6* (*79.6*–*94.0*)	*83.2* (*63.2*–*93.5*)	*5.27*^a^	*0.14*^a^
	*Lian et al. [*57*]*	*EP*^d^	*2018*	*MA* (*15*)	*NR*	*3309*	*NR*	*NR*	*Final diagnosis*	*85* (*84*–*87*)	*91* (*89*–*92*)	*8.94* (*5.64*–*14.18*)	*0.14* (*0.08*–*0.26*)
	Koh et al. [75]	EP	2018	Singapore	20 h.	231	36	68	Final diagnosis	71.4 (60.5–80.8)	80.9 (72.5–87.6)	3.73 (2.50–5.57)	0.35 (0.25–0.50)
	*Maw et al. [*58*]*	*EP*^d^	*2019*	*MA* (*6*)	*NR*	*1827*	*20*–*62*	*NR*	*Final diagnosis or echoc./BNP*	*88* (*75*–*95*)	*90* (*88*–*92*)	*8.63* (*6.93*–*10.74*)	*0.14* (*0.06*–*0.29*)
	*Staub et al. [*59*]*	*EP*^d^	*2019*	*MA* (*14*)	*NR*	*2778*	*24*–*88*	*NR*	*Final diagnosis*	*NR* (*75*–*90*)^b^	*NR* (*80*–*90*)^b^	*NR*	*NR*
	Pivetta et al. [60]	EP	2019	Italy	40 *x*	518	43	79	Final diagnosis	93.5 (87.7–97.2)	95.5 (90.5–98.3)	20.9 (9.54–45.7)	0.07 (0.03–0.13)
	Bekgoz et al. [76]	EP	2019	Turkey	2 h.	383	22	66	Final diagnosis	87 (79–93)	97 (94–98)	29^a^	0.13^a^
Pneumonia (adults)	*Ye et al. [*63*]*	*EP*	*2015*	*MA* (*5*)	*NR*	*742*	–	*NR*	*Final diagnosis*	*95* (*93*–*97*)	*90* (*86*–*94*)	*9.5*^a^	*0.056*^a^
	*Orso et al. [*62*]*	*EP*	*2018*	*MA* (*17*)	*NR*	*5108*	*NR*	*67*	*Final diagnosis or CXR and/or CCT*	*92* (*87*–*96*)	*93* (*86*–*97*)	*13*^a^	*0,086*^a^
	*Staub et al. [*59*]*	*EP*^d^	*2019*	*MA* (*14*)	*NR*	*1896*	*30*–*85*	*NR*	*Final diagnosis or CXR and/or CCT*	*NR* (*85*–*95*)^b^	*NR* (*75*–*90*)^b^	*NR*	*NR*
	Amatya et al. [64]	EP	2018	Nepal	1 h.	62	71	59	CCT	91 (78–97)^a^	61 (36–83)^a^	2.34 (1.30–4.20)^a^	0.15 (0.05–0.41)^a^
	Koh et al. [75]	EP	2018	Singapore	20 h.	231	21	68	Final diagnosis	65.3 (50.4–78.3)	82.0 (74.9–87.8)	3.63 (2.44–5.40)	0.42 (0.29–0.63)
	Bekgoz et al. [76]	EP	2019	Turkey	2 h.	383	24	66	Final diagnosis	82 (78–89)	98 (97–99)	41^a^	0.18^a^
Pneumonia (children)	Copetti and Cattarossi [65]	EP	2008	Italy	NR	79	76	5	CXR, CT or final diagnosis	100^a^	100^a^	∞^a^	0^a^
	Shah et al. [66]	EP	2013	USA	1 h.	200	18	3	CXR	86 (71–94)	89 (83–93)	7.8 (5.0–12.4)	0.2 (0.1–0.4)
Pneumothorax	*Ebrahimi et al. [*70*]*	*EP*	*2014*	*MA* (*14*)^c^	–	*1803*	*NR*	*NR*	*CCT*	*88* (*82*–*94*)	*99* (*98*–*100*)	*88*^a^	*0.12*^a^
	*Staub et al. [*71*]*	*EP*^d^	*2018*	*MA* (*13*)	–	*2378*	*14*	*NR*	*CXR, CCT or chest tube* (*with rush of air*)	*81* (*71*–*88*)	*98* (*97*–*99*)	*67.9* (*26.3*–*148*)	*0.18* (*0.11*–*0.29*)
	Riccardi et al. [72]	EP	2019	Italy	–	190	9	59	CXR and/or CCT	94	100	∞^a^	0.06^a^
	Bekgoz et al. [76]	EP	2019	Turkey	2 h.	383	2	66	Final diagnosis	85	100	∞^a^	0.15^a^
COPD/Asthma	Koh et al. [75]	EP	2018	Singapore	20 h.	231	27	68	Final diagnosis	64.5 (51.3–76.3)	89.8 (83.4–94.3)	6.31 (3.72–10.72)	0.40 (0.28–0.56)
	Bekgoz et al. [76]	EP	2019	Turkey	2 h.	383	28	66	Final diagnosis	96 (90–97)	75 (70–80)	3.8^a^	0.05^a^

MA, meta-analysis (shown in italics with number of studies in brackets); Op., operators; Train., time spent in didactic intervention; h., hours; *x*, number of examinations; *n*, size of population; Prev., prevalence; Age, median or mean age in years; Sn., sensitivity; Sp., specificity; CI, confidence interval; LR+, positive likelihood ratio; LR− negative likelihood ratio; EP, emergency physician; NR, not reported; echoc., echocardiography; BNP, brain-type natriuretic peptide; CXR, chest X-ray; CCT, chest computed tomography

^a^Calculated by the authors

^b^The approximate overall 95% confidence interval based on the area under the curve

^c^EP sub-group analyzed separately

^d^The majority of studies included involved EPs

**Table 4:** Summary of test accuracy findings in vascular ultrasound

Test	Author	Op.	Year	Country (no. of studies in MA)	Train.	*n*	Prev (%)	Age	Criterion standard	Sn. in % (95% CI)	Sp. in % (95% CI)	LR+ (95% CI)	LR− (95% CI)
AAA (> 3 cm)—screening in general practice	Bravo-Merino et al. [90]	GP	2019	Spain	NR	76	17^b^/4.6^c^	70	Vascular surgical services ultrasound	100^b^/93.3 (75.4–99.9)^c^	100^b^/98.5 (94.3–100)^c^	∞^b^/62.2^c^	0^b^/0.07^c^
	Blois et al. [88]	GP	2012	Canada	NR	45	4.4	73	Radiologist	100 (15.8–100)^a^	100 (91.8–100)^a^	∞^a^	0^a^
	Bailey et al. [89]	GP	2001	USA	2 h.	79	5.1	NR	Radiologist	100 (39.8–100)^a^	100 (95.2–100)^a^	∞^a^	0^a^
AAA on clinical indication (cm)													
> 3	*Rubano et al. [*94*]*	*EP*	*2013*	*MA* (*7*)	*NR*	*655*	*23*	> *50*	*CT, MRI, radiologist US, aortography, surgical findings, autopsy*	*99* (*96*–*100*)	*98* (*97*–*99*)	*NR* (*10.8*–*∞*)	*NR* (*0*–*0.025*)
> 5	Lindgaard and Risgaard [93]	GP	2017	Denmark	2 d.	29	3	NR	Radiologist US	100 (2.5–100)^a^	100 (87.7–100)^a^	∞^a^	0^a^
DVT Mixed techniques (2-point, 3-point and duplex US)	*Pomero et al. [*97*]*	*EP*	*2013*	*MA* (*16*)	*10* *m.*–*6* *h.*	*2379*	*23*	*NR*	*Colour*-*flow duplex US by radiology or angiography*	*96.1* (*90.6*–*98.5*)	*96.8* (*94.6*–*98.1*)	*30.0* (*17.2*–*52.2*)	*0.04* (*0.02*–*0.10*)
DVT 2-point compression (CFV and PV)	*Lee* et al*. [*98*]*	*EP*	*2019*	*MA* (*9*)	*NR*	*1337*	*20*^a^	*49*–*73*	*Radiologist US*	*91* (*68*–*98*)	*98* (*96*–*99*)	*46*^a^	*0.09*^a^
	Torres-Macho et al. [99]	EP	2012	Spania	10 h.	76	35	NR	Radiologist US	92 (82–100)	98 (94–100)	46^a^	0.08^a^
	Mumoli et al. [96]	GP	2017	Italy	50 h.	1107	18	64	Vascular ultrasound physician experts	90.0 (88.2–91.8)	97.1 (96.2–98.1)	31.0^a^	0.10^a^
	Nygren et al. [101]	EP	2018	Sweden	45 m.	65	17	70	Radiologist US	100 (71.5–100)	90.7 (79.7–96.9)	10.8 (4.69–24.9)	0
DVT 3-point compression (CFV, SFV and PV)	*Lee et al. [*98*]*	*EP*	*2019*	*MA* (*8*)	*NR*	*1035*	*29*^a^	*47*–*68*	*Radiologist US and/or contrast venography*	*90* (*83*–*95*)	*95* (*83*–*99*)	*18*^a^	*0.11*^a^
	Crowhurst and Dunn [100]	EP	2013	Australia	2 h	178	14	57	Radiologist duplex US	77.8 (54.8–91.0)	91.4 (84.9–95.3)	9.04^a^	0.24^a^

MA, meta-analysis (shown in italics with number of studies in brackets); Op., operators; Train., time spent in didactic intervention; m., minutes; h., hours; d., days; *x*, examinations; *n*, size of population; Prev., prevalence; Age, median- or mean age; Sn., sensitivity; Sp., specificity; CI, confidence interval; LR+, positive likelihood ratio; LR−, negative likelihood ratio; GP, general practitioner; EP, emergency physician; NR, not reported; AAA, abdominal aortic aneurysm; DVT, deep vein thrombosis; CFV, common femoral vein; PV, popliteal vein; SFV, superficial femoral vein; CT, computed tomography; MRI, magnetic resonance imaging; US, ultrasound

^a^Only including the 20% that had the criterion standard applied

^b^By posterior probability distribution

^c^Calculated by the authors from available data

**Table 5:** Summary of test accuracy findings in abdominal ultrasound

Test	Author	Op.	Year	Country (no. of studies in MA)	Train.	*n*	Prev (%)	Age	Criterion standard	Sn. in % (95% CI)	Sp. in % (95% CI)	LR+ (95% CI)	LR− (95% CI)
*Hydronephrosis/nephrolithiasis*	*Wong et al. [*113*]*	*EP*	*2018*	*MA* (*9*)	*NR*	*1773*	*35*–*84*	*NR*	*CT, visualisation of stone or surgical findings*	*70.2* (*67.1*–*73.2*)	*75.4* (*72.5*–*78.2*)	*2.85*	*0.39*
	Javaudin et al. [114]	EP	2017	France	16 h.	50	38	47	Radiologist US	100 (82–100)	71 (52–86)	3.4 (2.0–6.0)	0
Pediatric hydronephrosis in UTI	Guedj et al. [115]	EP	2015	France	2 h.	382	5	9 m.	Radiologist US	76.5 (58.1–94.6)	97.2 (95.2–99.2)	27.3	0.25
Scrotal pathology	Blaivas et al. [118]	EP	2001	USA	NR	36	58	45	Radiologist colour doppler US	95 (78–99)	94 (72–99)	16^a^	0.053^a^
Cholelithiasis	Esquerrà et al. [121]	GP	2012	Spain	212 h.	115	56	NR	Radiologist US	88.9 (83.2–94.6)	100 (NR)	∞^a^	0.111
	Lindgaard and Risgaard [93]	GP	2017	Denmark	2 d.	62	42	NR	Radiologist US	92 (75–99)^a^	92 (78–98)^a^	11^a^ (3.7–33)^a^	0.08^a^ (0.02–0.32)^a^
	Scruggs et al. [125]	EP	2008	USA	–	575	60	NR	Radiologist US	88 (84–91)	87 (82–91)	6.8^a^	0.13^a^
	*Ross et al. [*124*]*	*EP*	*2011*	*MA* (*8*)	*NR*	*710*	*46*–*80*	*NR*	*Radiologist US, CT, MRI or surgical findings*	*89.8* (*86.4*–*92.5*)	*88* (*83.7*–*91.4*)	*7.5* (*NR*)	*0.12* (*NR*)
	Hilsden et al. [126]	EP	2018	Canada	Cert.	283	16	NR	Need for cholecystectomy	55 (40–70)	92 (88–95)	5.6^a^	0.49^a^
Cholelithiasis OR Cholecystitis	Schlager et al. [122]	EP	1994	Canada	NR	65	54	NR	Radiologist US or surgical findings	86 (70^a^–95^a^)	97 (83–100)^a^	26^a^ (4–177)^a^	0.15^a^ (0.07–0.33)^a^
Cholecystitis	Rosen et al. [127]	EP	2001	USA	NR	193	46	49	Clinical follow-up	92 (73–100)	78 (61–93)	4.2^a^	0.36^a^
	Summers et al. [128]	EP	2010	USA	NR	113	14	36	Surgical reports or clinical follow-up	87 (66–97)	82 (74–88)	4.7 (3.2–6.9)	0.16 (0.06–0.46)
	Shekarchi et al. [129]	EP	2018	Iran	4 h.	342	14	54	Radiologist US	89.58 (76.55–96.10)	96.59 (93.63–98.29)	4.30 (2.42–7.62)	0.017 (0.007–0.041)
	Tourghabe et al. [130]	EP	2018	Iran	NR	51	100	42	Surgical and pathology findings	37.84 (22.94–55.2)	100.0 (73.24–100.0)	∞	0.62 (0.48–0.80)
Appendicitis (pediatric)	*Benabbas et al. [*134*]*	*EP*	*2017*	*MA* (*4*)	–	*461*	*31*–*54*	*9*–*12*	*Final pathology*	*86* (*79*–*90*)	*91* (*87*–*94*)	*9.24* (*6.42*–*13.28*)	*0.17* (*0.09*–*0.30*)
	Nicole et al. [135]	EP	2018	Canada	2 d.	121	44	10	Pathology or clinical follow-up	53 (40–66)	82 (71–89)	2.94^a^	0.57^a^
Appendicitis (all ages)	*Lee and Yun [*136*]*	*EP*	*2019*	*MA* (*17*)	–	*2385*	*42*^a^	*6*–*37*	*Surgical or pathological findings*	*84* (*72*–*92*)	*91* (*85*–*95*)	*7.0* (*3.2*–*15.3*)	*0.22* (*0.12*–*0.42*)
Appendicitis (adults)	*Fields et al. [*137*]*	*EP*	*2017*	*MA* (*11*)^b^	–	*1621*	*NR*	*NR*	*CT, surgery, MRI or autopsy*	*80* (*76*–*83*)	*92* (*90*–*94*)	*10.2* (*8.2*–*12.7*)	*0.22* (*0.19*–*0.26*)
	Shahbazipar et al. [138]	EP	2018	Iran	8 h.	121	38	34	Pathology or clinical follow-up	63 (48–77)	99 (93–100)	63^a^	0.37^a^
	Sharif et al. [139]	EP	2018	Canada	NR	90	20	NR	Pathology, laparoscopy, CT and/or radiologist US	69.2 (48.1–84.9)	90.6 (80.0–96.1)	7.4 (3.3–16.5)	0.3 (0.2–0.6)
	Corson-Knowles and Russell [140]	EP	2018	USA	20 m	76	37	27^a^	Pathology results or clinical follow-up	42.8 (25.0–62.5)	97.9 (87.5–99.8)	20.6 (2.8–149.9)	0.58 (0.42–0.80)
Intussusception (pediatric)	Riera et al. [141]	EP	2012	USA	1 h.	82	16	24 m.	Radiologist US	85 (54–97)	97 (89–99)	29 (7.3–117)	0.16 (0.04–0.57)
	Lam et al. [142]	EP	2014	USA	1 h.	44	23	31	Radiologist study	100 (66–100)	97 (82–100)	32 (4.65–220)	0
Small bowel obstruction	Unlüer et al. [146]	EP	201	Turkey	6 h.	174	49	56	Surgical findings, CT or clinical follow-up	97.7 (94.5–100)	92.7 (87.0–98.3)	13.4 (6.2–28.9)	0.025^a^
	Jang et al. [147]	EP	2011	USA	10 m.	76	43	NR	Abdominal CT	91 (75–98)	84 (69–93)	5.6 (2.8–11.1)	0.1 (0.04–0.3)
	Frasure et al. [148]	EP	2018	USA	NR	47	68	63	Abdominal CT	93.8 (79.2–99.2)	93.3 (68.1–99.8)	14.1 (2.11–93.6)	0.07 (0.02–0.26)
	Becker et al. [145]	EP	2019	USA	30 m.	217	43	55	Abdominal CT	88 (80–94)	54 (45–63)	1.92 (1.56–2.35)	0.22 (0.12–0.39)
Ascites	Lindgaard and Riisgaard [93]	GP	2017	Denmark	2 d.	34	9	NR	Radiologist US	100 (29–100)^a^	100 (89–100)^a^	∞	0
Constipation in children	Doniger et al. [149]	EP	2018	USA	1.5 h.	50	64	10 ± 4	Rome III questionnaire	86 (67–95)	71 (53–85)	3.0^a^	0.20^a^

MA, meta-analysis (shown in italics with number of studies in brackets); Op., operators; Train., time spent in didactic intervention; m., minutes; h., hours; d., days; *n*, size of population; Prev., prevalence; Age, median- or mean age; Sn., sensitivity; Sp., specificity; CI, confidence interval; LR+, positive likelihood ratio; LR−, negative likelihood ratio; GP, general practitioner; EP, emergency physician; NR, not reported; CT, computed tomography; MRI, magnetic resonance imaging; US, ultrasound

^a^Calculated by the authors from available data

^b^EP sub-group analyzed separately

**Table 6:** Summary of test accuracy findings in ocular ultrasound

Test	Author	Op.	Year	Country (no. of studies in MA)	Train.	*n*	Prev (%)	Age	Criterion standard	Sn. in % (95% CI)	Sp. in % (95% CI)	LR+ (95% CI)	LR− (95% CI)
Retinal detachment	*Gottlieb et al. [*168*]*	*EP*	*2019*	*MA* (*5*)^b^	*0.5*–*2* *h.*	*455*^a^	*12*–*38*	*46*–*60*	*Orbital CT* (*1*) *or ophthalmology evaluation* (*6*)	*93.9* (*78.7*–*98.5*)	*92.4* (*85.6*–*96.1*)	*12.4*^a^	*0.0660*^a^
	Jacobsen et al. [169]	EP	2016	USA	0.5 h.	109	31	49	Ophthalmology evaluation	91 (76–98)	96 (89–99)	23^a^	0.094^a^
	Lahham et al. [170]	EP	2019	USA	1 h.	225	21	51	Ophthalmology evaluation	96.9 (80.6–99.6)	88.1 (81.8–92.4)	8.14^a^	0.0352^a^
	Ojaghihaghighi et al. [171]	EP	2019	Iran	16 h.	351	8	34	Ophthalmology evaluation	88.9 (70.8–97.6)	100.0 (98.9–100.0)	∞	0.11 (0.038–0.32)
Vitreous haemorrhage	Lahham et al. [170]	EP	2019	USA	1 h.	225	24	51	Ophthalmology evaluation	81.9 (63.0–92.4)	82.3 (75.4–87.5)	4.63^a^	0.220
	Ojaghihaghighi et al. [171]	EP	2019	Iran	16 h.	347	13	34	Ophthalmology evaluation	97.8 (88.2–99.9)	98.7 (96.7–99.6)	74.8 (28.2–198.0)	0.023 (0.032–0.16)
Vitreous detachment	Lahham et al. [170]	EP	2019	USA	1 h.	225	15	51	Ophthalmology evaluation	42.5 (24.7–62.4)	96.0 (91.2–98.2)	10.6^a^	0.599^a^
Lens dislocation	Ojaghi Haghighi et al. [172]	EP	2014	Iran	NR	130	10	35	Orbital CT	84.6 (53.7–97.3)	98.3 (93.3–99.7)	49.5 (12.3–199.4)	0.15 (0.04–0.56)
	Ojaghihaghighi et al. [171]	EP	2019	Iran	16 h.	348	9	34	Orbital CT	96.8 (83.3–99.9)	99.4 (97.8–99.9)	154.8 (38.8–617.0)	0.032 (0.005–0.22)
						346	9		Ophthalmology evaluation	96.6 (82.2–99.9)	98.8 (96.9–99.7)	77.7 (29.3–206.0)	0.035 (0.0051–0.24)
Globe foreign body	Ojaghihaghighi et al. [171]	EP	2019	Iran	16 h.	350	5	34	Orbital CT	100.0 (79.4–100.0)	99.7 (98.3–100.0)	335.0 (47.3–2,371.0)	0
Globe rupture (except clinically obvious)	Ojaghihaghighi et al. [171]	EP	2019	Iran	16 h.	350	1	34	Orbital CT	100.0 (39.7–100.0)	99.7 (98.4–100.0)	347.0 (49.0–2,456.0)	0
Retrobulbar haematoma	Ojaghihaghighi et al. [171]	EP	2019	Iran	16 h.	350	9	34	Orbital CT	95.7 (78.1–99.9)	99.7 (98.3–100.0)	313.7 (44.2–2225.0)	0.044 (0.0064–0.30)

MA, meta-analysis (shown in italics with number of studies in brackets); Op., operators; Train., time spent in didactic intervention; h., hours; *n*, size of population; Prev., prevalence; Age, median- or mean age; Sn., sensitivity; Sp., specificity; CI, confidence interval; LR+, positive likelihood ratio; LR−, negative likelihood ratio; EP, emergency physician; NR, not reported; CT, computed tomography

^a^Calculated by the authors from available data

^b^EP sub-group analyzed separately

**Table 7:** Summary of test accuracy findings in soft-tissue ultrasound

Test	Author	Op.	Year	Country (no. of studies in MA)	Train.	*n*	Prev (%)	Age	Criterion standard	Sn. in % (95% CI)	Sp. in % (95% CI)	LR+ (95% CI)	LR− (95% CI)
Abscess	*Barbic et al. [*173*]*	*EP*	*2017*	*MA* (*8*)	*15* *m.*–*1 d.*	*747*	*NR*	*All*	*Positive I&D and/or follow*-*up*	*96.2* (*91.1*–*98.4*)	*82.9* (*60.4*–*93.9*)	*5.63* (*2.2*–*14.6*)	*0.05* (*0.01*–*0.11*)
	*Subramaniam et al. [*174*]*	*EP*	*2016*	*MA* (*6*)	*30* *m.*–*2 d.*	*413*^*a*^	*NR*	*All*	*Positive I&D and/or follow*-*up*	*97* (*94*–*98*)	*83* (*75*–*88*)	*5.5* (*3.7*–*8.2*)	*0.04* (*0.02*–*0.08*)
	Gaspari et al. [175]	EP	2012	USA	NR	65	46	42	*Positive I&D and/or follow*-*up*	96.7 (87.9–99.4)	85.7 (77.4–88.0)	6.76^a^	*0.0385*^a^
	Mower et al. [177]	EP	2019	USA	NR	1216	68	36	Positive I&D immediately or 1 week	94.0 (92.1–95.4)	94.1 (91.3–96.2)	15.9 (10.7–23.6)	0.06 (0.05–0.08)
Peritonsillar abscess	Costantino et al. [182]	EP	2012	USA	NR	14	57	26	Positive I&D and follow-up	100 (63–100)^a^	100 (54–100)^a^	∞^a^	0^a^
Dental abscess	Adhikari et al. [183]	EP	2012	USA	NR	19	63	40	Positive I&D	92 (62–100)^a^	100 (59–100)^a^	∞^a^	0.08 (0.01–0.54)
Foreign body	Friedman et al. [186]	EP	2005	USA	NR	131	9	10	Identification of FB	66.7 (34.8–90.1)	96.6 (91.6–99.1)	19.8^a^ (6.99–56.3)^a^	0.34^a^ (0.15–0.77)^a^

MA, meta-analysis (shown in italics with number of studies in brackets); Op., operators; Train., time spent in didactic intervention; m., minutes; d., days; *n*, size of population; Prev., prevalence; Age, median- or mean age; Sn., sensitivity; Sp., specificity; CI, confidence interval; LR+, positive likelihood ratio; LR−, negative likelihood ratio; EP, emergency physician; NR, not reported; I&D, incision and drainage

^a^Calculated by the authors from available data

**Table 8:** Summary of test accuracy findings in musculoskeletal ultrasound

Test	Author	Op.	Year	Country (no. of studies in MA)	Train.	*n*	Prev (%)	Age	Criterion standard	Sn. in % (95% CI)	Sp. in % (95% CI)	LR+ (95% CI)	LR− (95% CI)
Ulnar ligamentous strain injuries	Lee and Yun [188]	EP	2018	South Korea	1 week	65	NR	31	MRI	97.2 (92.0–99.4)	96.8 (93.5–98.7)	30.4^a^	0.03^a^
	Oguz et al. [187]	EP	2017	Turkey	NR	80	19	46	MRI	66.7 (41.7– 84.8)	100 (94.4–100)	∞^a^	0.333^a^
Ankle anterior talofibular ligament strain injury	Gün et al. [189]	EP	2013	Turkey	6 h.	65	49	34	MRI	93.8 (79.2–99.2)	100 (89.4–100)	∞	0.06
	Lee and Yun [190]	EP	2017	South Korea	1 week	85	77	27	MRI	98.5 (91.7–100)	95.0 (75.1–99.9)	19.7^a^	0.0158^a^
Ankle calcaneofibular ligament injury	Lee and Yun [190]	EP	2017	South Korea	1 week	85	21	27	MRI	96.4 (81.7–99.9)	100 (81.5, 100)	∞^a^	0.0360^a^
Achilles tendon rupture	Lee and Yun [190]	EP	2017	South Korea	1 week	85	8	27	MRI	100 (59.0–100)	100 (95.4–100)	∞^a^	0^a^
Hip effusion (paediatric)	Cruz et al. [194]	EP	2018	USA	NR		926	6	Final diagnosis	85 (79–89)	98 (96–99)	43^a^	0.15^a^
	Vieira and Levy [193]	EP	2010	USA	30 min	55	43	8	Radiologist performed US	80 (51–95)	98 (85–99)	32^a^ (4.5–225)^a^	0.21^a^ (0.07–0.57)^a^
Extremity tendon laceration	Wu et al. [191]	EP	2012	USA	2 h.	34	38	> 16	Wound exploration or MRI	100 (75–100)^a^	95 (76–100)	20^a^	0^a^
	Mohammadrezai et al. [192]	EP	2017	Iran	6 h.	60	30	30	Wound exploration	94.4 (72.7–99.8)	100.0 (91.5–100.0)	∞^a^	0.06 (0.01–0.37)
Shoulder dislocation	*Gottlieb* et al. [196]	*EP*	*2019*	*MA* (*7*)	–	*739*	*12*–*60*	*35*	X-ray	*99.1* (*84.9*–*100*)	*99.9* (*88.9*–*100*)	*796.2* (*8.0*–*79,086*)	*0.01* (*0*–*0.17*)
ACL or PCL ruptures	Lee and Yun [197]	EP	2019	South Korea	1 week	62	73	29	MRI	92.2 (81.1–97.8)	95.9 (88.5–99.1)	22.5^a^	0.0813^a^
Skull fractures (paediatric)	Weinberg et al. [198]	EP	2010	USA	1 h.	21	10	NR	CT	100 (20–100)	100 (79–100)	∞	0
	Riera and Chen [199]	EP	2012	USA	NR	46	24	2	CT	82 (48–97)	94 (79–99)	14^a^	0.085^a^
	Parri et al. [200]	EP	2013	Italia	1 h.	55	64	3.7	CT	100 (88.2–100)	95.0 (75.0–99.9)	20^a^	0^a^
	Rabiner et al. [201]	EP	2013	USA	30 m.	69	12	6.4	CT	88 (53–98)	97 (89–99)	27 (7–107)	0.13 (0.02–0.81)
	Choi et al. [202]	EP	2018	South Korea	1 h.	87	15	21 months	CT	76.9 (46.0–93.8)	100 (93.9–100)	∞^a^	0.231^a^
	Parri et al. [203]	EP	2018	Italia	NR	115	84	8 months	CT	90.9 (82.9–96.0)	85.2 (66.3–95.8)	6.14^a^ (2.48–15.2)^a^	0.11^a^ (0.05–0.21)^a^
Clavicle	Cross et al. [204]	EP	2010	USA	NR	100	43	11	X-ray	95 (83–99)	96 (87–99)	27 (7–106)	0.05 (0.01–0.19)
	Weinberg et al. [198]	EP	2010	USA	1 h.	15	60	NR	X-ray	89 (51–99)	83 (36–99)	5.3 (0.87–32.4)	0.13 (0.01–0.90)
	Chien et al. [205]	EP	2011	USA	15 m.	58	67	7	X-ray	89.7 (75.8–97.1)	89.5 (66.9–98.7)	8.53	0.11
Elbow fractures (paediatric)	*Lee and Yun [*206*]*	*EP*	*2019*	*MA* (*5*)^b^	*NR*	*445*	*44*	*6*–*9*	X-ray	*95* (*87*–*100*)	*94* (*88*–*100*)	*16*^a^	*0.053*
Forearm fractures (paediatric)	*Chartier et al. [*208*]*	*EP*^*c*^	*2017*	*MA* (*10*)	*NR*	*NR*	*NR*	*NR*	X-ray	*93.1* (*87.2*–*96.4*)	*92.9* (*86.6*–*96.4*)	*14.1* (*6.71*–*25*)	*0.08* (*0.04*–*0.15*)
	Hedelin et al. [209]	EP	2017	Sweden	1.5 h.	116	65	11	X-ray	97.4 (90.9–99.7)	84 (67.2–94.7)	6.23^a^ (2.78–13.96)^a^	0.03^a^ (0.01–0.12)^a^
	Rowlands et al. [210]	EP	2017	Australia	3.5 h.	419	56	9	X-ray	91.5 (87.1–94.7)^a^	87.5 (81.9–92.0)^a^	7.36^a^ (5.01–10.8)^a^	0.10^a^ (0.06–0.15)^a^
Distal forearm fractures (all ages)	*Douma*-*den Hamer et al. [*211*]*	*EP*^*c*^	*2016*	*MA* (*16*)	*NR*	*1204*	*53*	*NR*	X-ray	*97* (*93*–*99*)	*95* (*89*–*98*)	*20* (*8.5*–*47.2*)	*0.03* (*0.01*–*0.08*)
	Sivrikaya et al. [212]	EP	2016	Turkey		93		47	CT/X-ray and orthopaedic evaluation	97.4 (90.2e99.5)	92.6 (85.5–96.5)	13.1 (6.7–25.6)	0.02 (0–0.10)
Wrist fracture (any)	Oguz et al. [187]	EP	2017	Turkey	NR	80	80	46	X-ray and/or CT	95.31 (87.10–98.39)	93.75 (71.67–98.89)	15.25^a^ (2.28–101)^a^	0.05^a^ (0.02–0.15)^a^
Metacarpal or phalangeal fractures	Tayal et al. [213]	EP	2007	USA	NR	78	40	34	X-ray or surgical findings	90 (74–97)	98 (95–100)	42.5 (NR)	0.1 (NR)
	Neri et al. [214]	EP^d^	2014	Italy	2 h.	153	39	12	X-ray	91.5 (84.4–98.7)	96.8 (93.2–100)	28.7^a^ (9.39–87.5)^a^	0.09^a^ (0.04–0.20)^a^
Metacarpal fractures	Kozaci et al. [215]	EP	2015	Turkey	NR	66	55	24	X-ray (reported by EPs)	92 (NR)	87 (NR)	7.1^a^	0.091^a^
	Kocaoğlu et al. [219]	EP	2016	Turkey	0	96	40	30	X-ray (reported by EP)	92.5 (78.5–98.0)	98.3 (89.5–99.9)	54.4^a^	0.08^a^
Fifth metacarpal fracture	Aksay et al. [216]	EP	2015	Turkey	1 h.	81	48	28	X-ray (reported by OS) or CT	97.4 (84.9–99.9)	92.9 (79.4–98.1)	14 (4.58–41)	0.03 (0.00–0.19)
Proximal or middle phalanx fracture	Aksay et al. [217]	EP	2016	Turkey	NR	119	24	27	X-ray (reported by OS)	79.3 (59.7–91.2)	90 (81.4–95)	7.93 (4.15–15)	0.23 (0.11–0.47)
Distal phalanx fracture	Gungor et al. [218]	EP	2016	Turkey	3 h.	45	29	32	X-ray reported by EP	100 (79–100)	98 (91–100)	59 (8–412)	0
Nail bed injury	Gungor et al. [218]	EP	2016	Turkey	3 h.	45	73	32	Visual inspection	93 (80–99)	100 (74–100)	∞	0.06 (0.02–0.23)
Tibia and/or fibula (anywhere)	Kozaci et al. [220]	EP	2017	Turkey	2 h.	62	34	5–55	X-ray reported by EP	100 (84–100)^a^	93 (80–98)^a^	13.7^a^ (4.60–40.6)^a^	0^a^
Medial or lateral malleolus fracture	*Chartier* et al*. [*208*]*	*EP*^*c*^	*2017*	*MA* (*4*)	*NR*	*609*^*a*^	*7*–*23*	*Adults*	*X*-*ray and/or CT*	*89.5* (*77.0*–*95.6*)	*94.2* (*86.1*–*97.7*)	*16.4* (*6.57*–*33.5*)	*0.12* (*0.05*–*0.24*)
Lateral malleolus	Ozturk et al. [223]	EP	2018	Turkey	2 h.	120	35	41	X-ray and/or CT evaluated by OS	100 (90–100)	93 (85–98)	8.4 (3.6–19.3)	0
Malleolar fracture OR fifth metatarsal fracture	Tollefson et al. [222]	EP	2016	USA	1 h.	50	36	35	X-ray reported by radiologist	100 (78–100)	100 (87–100)	∞^a^	0^a^
Navicular fracture	Atilla et al. [221]	EP	2014	Turkey	4 h.	34	15	37	X-ray and/or CT evaluated by OS	40 (7–83)	93 (76–99)	5.7^a^	0.65^a^
Fifth metatarsal fracture	Atilla et al. [221]	EP	2014	Turkey	4 h.	97	30	37	X-ray and/or CT evaluated by OS	100 (85–100)	96 (87–99)	25^a^	0^a^
	Yesilaras et al. [224]	EP	2014	Turkey	0	84	41	36	X-ray reported by OS	97.1 (82.9–99.8)	100 (91.1–100)	∞	0.03 (0.01–0.21)
	Kozaci et al. [225]	EP	2017	Turkey	2 h.	72	39	5–55	X-ray reported by EP	93 (77–99)^a^	89 (75–96)^a^	8.17^a^ (3.56–18.7)^a^	0.08^a^ (0.02–0.31)^a^

MA, meta-analysis (shown in italics with number of studies in brackets); Op., operators; Train., time spent in didactic intervention; m., minutes; h., hours; *n*, size of population; Prev., prevalence; Age, median- or mean age; Sn., sensitivity; Sp., specificity; CI, confidence interval; LR+, positive likelihood ratio; LR−, negative likelihood ratio; EP, emergency physician; NR, not reported; CT, computed tomography; MRI, magnetic resonance imaging; US, ultrasound; OS, orthopaedic surgeon

^a^Calculated by the authors from available data

^b^EP sub-group analyzed separately

^c^The majority of studies included involved EPs

To the extent any other parameters than diagnostic accuracy were studied, this is presented narratively in the below text.

### Heart

Studies on indications relating to the heart are summarized in Table 2. Even though a GP in a Norwegian pilot study from 1985 concluded that “echocardiography will not have any diagnostic significance in general practice in the foreseeable future” [31], a similar UK study was more positive in 1998 where one found GP performed evaluation of left-ventricular function frequently altered management [32].

Three studies from the last few years evaluated GPs’ use of echocardiography compared to cardiologist as the reference, all of which found that, after 4–28 h of instruction, the GP could assess left-ventricular form and function with an accuracy high enough to impact management [33–35]. GPs have been found to reliably measure the mitral annular plane systolic excursion (MAPSE) through the use of pocket ultrasound after an 8 h teaching program with a sensitivity of 83% and a specificity of 78% [33]. A Spanish study found high accuracy for detecting left-ventricular hypertrophy (LVH) with GP operated pocket ultrasound in hypertensive patients in general practice, with a LR+ of 56 and a LR− of 0.1 [34]. They also found clinically useful test accuracy for other abnormalities. Another Spanish study found that GPs using pocket echocardiography on several indications had a very high specificity (93–100%) for a range of diagnoses, including LVH and valvular pathologies, but a rather low sensitivity (41–72%) [35].

Nine studies showed that EPs of varying experience could estimate left-ventricular ejection fraction (LVEF) and showed an overall agreement with cardiologists of between 84 and 93%, both on visual estimation and calculated values using, e.g., E-point septal separation [36–44]. Another study showed good agreement between EPs and cardiac sonographers on obtaining windows for left-ventricular outflow tract for velocity time integral studies [45], and it has been shown that EPs were able to obtain those windows for more than half of their ED patients [46]. Three studies identified high sensitivities and moderate-to-very good agreement with cardiologists for detection of diastolic dysfunction [47–49], while an Italian study found a high correlation between EP findings of restrictive mitral pattern and the presence of left-ventricular heart failure, with an LR+ of 8.27 [50]. EPs have also been shown to have good inter-rater agreement for the assessment of overall diastolic function [51].

Emergency physicians ability to detect wall motion abnormalities showed very good agreement with cardiologists in two studies [43, 52], while a 2018 US study sought to find whether EPs could use speckle tracking software to identify wall motion abnormalities and found that the sensitivity was low at 29%, but specificity high at 88% [53].

The ability to detect pericardial fluid by EPs was studied in four studies which all found sensitivities from 60 to 96% and specificities from 96 to 100% despite short training periods. False-negative findings were more likely for smaller effusions [39, 42, 43, 54].

### Lungs

Findings from studies on lung ultrasound are detailed in Table 3. Lung ultrasound (LUS) can be used to detect diffuse interstitial syndrome (bilateral B lines), which, in the setting of suspected acute decompensated heart failure (ADHF), likely signifies pulmonary oedema. We identified five meta-analyses on this utility of LUS in the emergency department, all concluding that both the sensitivity and specificity are very high [55–59], and indeed the one test with the best test characteristics compared to all other clinical parameters for ADHF ever studied [55]. One meta-analysis only included studies where also chest X-ray (CXR) had been compared with LUS towards the same gold standard, and found that CXR had the same specificity (90%) but lower sensitivity than LUS (73% vs 88%) [58]. A recent randomised-controlled study by Pivetta et al. [60], not analyzed in these meta-analyses, allocated patients after the initial suspicion of ADHF into groups receiving CXR and pro-brain natriuretic peptide (pro-BNP) or LUS, and found not only that LUS had both superior specificity and sensitivity compared to the criterion standard of final chart diagnosis, but also a shorter time to the diagnosis (5 min vs 104.5 min). Finally, one Australian study analyzed inter-rater agreement between experienced and novice EP lung sonographers which was found to be good, with a Cohen’s kappa coefficient of 0.70 [61].

Three meta-analyses were identified that assessed the accuracy of LUS in diagnosing pneumonia in unselected adult populations [59, 62, 63]. Orso et al. found 17 studies in ED populations where focal subpleural consolidations, focal B lines, or a combination of these were considered a positive finding, using X-ray and/or CT as the criterion standard, and found a pooled sensitivity of 92% and a specificity of 93%, similar to the findings in the meta-analysis by Staub et al. [59]. Ye et al. [63] only included studies where LUS was directly compared to CXR using the final diagnosis as the criterion standard, and found that LUS had a sensitivity of 95% against 77% for CXR, while the specificity was the same, 90%. A recent study not included in these meta-analyses found a similar superiority to CXR in a Nepalese ED population [64].

An Italian study on PoCUS for pneumonia in a paediatric population by one expert EP (*n* = 79) agreed with the final diagnosis of pneumonia in all cases and had no false-positive findings [65]. A later study in 200 children with suspected pneumonia (prevalence = 18%) showed sensitivity and specificity of 86% and 89%, respectively, when compared to CXR as the gold standard [66]. Ultrasound has been shown to be more sensitive than CXR in a study of a paediatric ED population, but less specific [67], and another study showed a 39% reduction in use of CXR for the final diagnosis of pneumonia in children in a randomised trial, with no cases of missed diagnoses or complications [68]. PoCUS by paediatric EPs instead of CXR was in one study associated with less time spent and decreased overall costs [69].

The absence of pleural sliding and B lines is a sign of pneumothorax, and finding the point where the pleural layers separates from each other, the lung point, is pathognomonic. A recent meta-analysis showed a very high accuracy of PoCUS when performed by EPs, with 88% sensitivity and 99% specificity, and it was superior to CXR which had 46% sensitivity and 100% specificity [70]. The findings were similar in another recent meta-analysis, albeit with a somewhat heterogeneous operator group [71], as well as in a recent original prospective observational study [72].

Two studies from 2017 used the total cases of positive findings of rib fractures found by either LUS or CXR as the criterion standard (assuming that there were no false-positive findings) and found a sensitivity of 81–98% in LUS compared to 41–53% for CXR [73, 74]. A third study found a similar concordance between LUS and CXR and/or CCT [72].

Two studies evaluated the accuracy of PoCUS through present lung sliding and predominant A lines as a marker for asthma or chronic obstructive pulmonary disease (COPD) in the setting of dyspnoea, and found an LR+ of 3.8–6.3 and an LR− of 0.05–0.40 [75, 76]. Such LUS findings can also be seen in patients without pulmonary pathology, which may explain the poorer test characteristics seen in the undifferentiated ED populations compared to what has been seen in intensive-care unit populations [59].

Finally, we identified 11 articles which studied the impact of different PoCUS protocols on the overall diagnosis of patients presenting with undifferentiated respiratory or chest symptoms. An Italian ED-based study showed that LUS in the setting of pleuritic pain without dyspnoea had 97% sensitivity and 96% specificity for detecting lesions that did not show up on CXR, using other imaging modalities and final diagnosis as their criterion standard [77]. Another Italian study found that LUS in dyspnoeic patients changed the diagnosis in 44% of cases and altered management in 58% [78]. Danish EPs evaluating dyspnoeic patients with PoCUS of heart, lung, and deep veins found life-threatening diagnoses that were missed in the primary assessment in 14% of patients, reporting a total of 100% sensitivity and 93% specificity for the diagnosis of such conditions [79]. The same group randomised 320 dyspnoeic patients (and SpO2 < 95%) into a PoCUS group or management as usual, and found as their primary endpoint a significant 24% higher accuracy in diagnosis at 4 h (88% vs 64%), using masked audit as the gold standard [80]. Similarly, two studies found a significant reduction in time needed for diagnosis using integrated ultrasound on dyspnoeic patients [81, 82]. It has also been shown that the addition of heart and lung PoCUS allowed the EPs to reduce the number of diagnoses on their differential diagnosis list from 5 to 3 (*p* < 0.001) [83], and also three other studies showed statistical significance in PoCUS overall diagnostic accuracy in patients with dyspnoea [84–86]. One USA study could not show significant diagnostic or management changes when a PoCUS protocol was applied to dyspnoeic patients in ED significantly, but it improved EPs’ confidence levels [87].

### Vessels

Main test characteristic findings can be found in Table 4.

Screening for abdominal aortic aneurysms (AAA) by GPs would require a very high accuracy to avoid false positive in a relatively low pre-test probability population, even if one selects the population who is at risk, men who have smoked in the ages between 65 and 75. We identified three small studies of GPs’ screening for AAA in such populations against a gold standard [88–90]. All found 100% accuracy for AAA greater than 3 cm and concluded screening by GPs were feasible. One larger feasibility study only confirmed positive cases [91]. Hoffmann et al. [92] also found screening by EPs in the emergency department feasible, but requiring substantial resources for a low success rate.

In a Danish study, inexperienced GPs achieved 100% accuracy for AAA > 5 cm compared to radiologists when the scan was performed on clinical indication [93]. Similarly, one meta-analysis showed that EPs have very high accuracy for detecting AAA > 3 cm compared to formal radiologist performed ultrasound when performed on indication [94].

One Japanese retrospective study investigated the impact of GPs screening of carotid intima media thickness in patients at risk of coronary artery disease (CAD) on later interventions, and found an increase in the prevalence of CAD in patients referred to a local specialist centre and higher probability of coronary angiograms and revascularization [95].

One multi-centre study assessed Italian GPs’ accuracy of a two-point compression technique for the identification of lower extremity deep vein thrombosis (DVT) and found 90% sensitivity and 97% specificity compared to radiologist ultrasound [96]. A meta-analysis on EPs use of PoCUS for detection of DVT found even higher accuracy with a sensitivity of 96% and a specificity of 97% [97]. A newer meta-analysis from 2019 shows a pooled sensitivity of 91% and a specificity of 98% for the two-point compression technique (assessing the common femoral vein and the popliteal vein) and similarly 90% and 95% for the three-point compression technique (including the superficial femoral vein) [98]. Three other studies not analyzed in above meta-analyses show similar test accuracies [99–101]. One study showed a > 4-fold reduction in ED length of stay for the group with EP-performed DVT studies vs the radiology department patients [102].

Ultrasound-guided peripheral venous catheter (PVC) insertion has been shown in some studies to reduce time and attempts [103–105], while others show similar or even worse success rate [106–108]. One study found that ultrasound-guided PVC insertion was associated with a higher rate of extravasation, 3.6% vs 0.3% [109]. Another study showed a 73% success of cannulation of the brachial or the basilic vein after two failed attempts without ultrasound, but also showed an 8% rate of extravasation at 1 h [110]. One group evaluated EPs use of PoCUS before peripheral venous cannulation of children less than 7 years before cannulation as usual, and found visible veins on ultrasound a strong predictor for successful cannulation [111]. It has also been found that EPs could insert a standard 2.5-in., 18-gauge peripheral venous catheter in the internal jugular vein with a success rate of 97.1% after two failed attempts by management as usual by nursing staff [112].

### Abdomen

The main findings on diagnostic test accuracy of abdominal PoCUS are listed in Table 5.

One meta-analysis of EPs’ findings of hydronephrosis as a surrogate for nephrolithiasis in patients presenting with renal colic found only moderate sensitivity and specificity [113]. Moderate-to-severe hydronephrosis is highly specific for the presence of a stone at 94%, but only with a sensitivity of 29%. One study not included in this meta-analysis found 100% sensitivity, but moderate specificity [114]. A French study found that EPs correctly identified hydronephrosis in children with urinary tract infections (prevalence = 5%) with a sensitivity of 76.5% and a specificity of 97.2% [115]. Finally, one large (*n* = 2759) study, randomising patients into diagnosis through EP PoCUS, radiologist ultrasound or computed tomography (CT), found no difference in high-risk diagnoses that could be due to missed or delayed diagnosis after 30 days, and showed overall lower cumulative radiation exposure at 6 months for both ultrasound groups compared to the CT group [116]. They also showed a slight, but significant, reduction in ED length of stay, while another study found halving of the length of stay [117].

Only one small, retrospective study reviewed EPs diagnostic accuracy of scrotal PoCUS, and found that the EPs correctly diagnosed epididymitis, orchitis, and testicular torsion in 35 of 36 cases [118]. No cases of testicular torsion were missed.

Two Norwegian studies demonstrated clinical usefulness for the use of GP operated PoCUS to demonstrate cholelithiasis already in the 80s [119, 120], and also a more recent study shows high agreement between GP and radiologist performed ultrasound [121]. In the ED setting, a high accuracy was shown already in a 1994 study [122] and Blaivas et al. [123] showed a significant reduction in the length of stay in the emergency department when EPs used PoCUS for diagnosis of biliary disease. One meta-analysis found an LR+ of 7.5 and LR− of 0.12 on EP-performed PoCUS for cholelithiasis [124], similar to a large, retrospective study not included in the meta-analysis [125]. A similar high specificity was found in a more recent study, and a sensitivity of 55% when using eventual need for cholecystectomy as their gold standard [126]. When it comes to cholecystitis, the LR+ ranged from 4.2 to 4.7 and the LR− from 0.05 to 0.39 in three studies of varying design [127–129]. Summers et al. [128] found that there were close agreement with radiology department ultrasound when compared to the criterion standard of surgical reports and follow-up, and suggested that patients with negative EP scans are unlikely to require surgery. Another study could not conclude the same, as they, in contrast to the other studies, only found 38% sensitivity using surgical findings as the criterion standard [130]. The positive likelihood ratio was high nevertheless, as specificity in their study was 100%. A Turkish study found that diagnosis and management were more likely to be affected if the clinician had moderate, rather than low or high, suspicion about the diagnosis prior to the study [131]. One study performed PoCUS on patients presenting with non-traumatic epigastric pain, and found a cholelithiasis prevalence of 39% in this population, even though the treating EP did not initially consider the need for biliary ultrasound in 85% of these cases [132]. A USA study found that the presence of a dilated common bile duct on EP-performed PoCUS, in the absence of laboratory findings or signs of cholecystitis on ultrasound, was unlikely to be a good indicator for complicated biliary pathology (sensitivity 23.7% and specificity 77.9%) [133].

Appendicitis has several hall-mark findings such as oedematous wall and overall thickness. One meta-analysis found an LR+ of 9.24 on EP-performed ultrasound for appendicitis in children [134], reproduced in one study published since [135]. Lee and Yun [136] found LR+ of 7.0 in a 2019 meta-analysis of PoCUS on all ages, while Fields et al. [137] found LR+ of 10.2 in their sub-group analysis of EP-performed PoCUS for appendicitis in a 2017 meta-analysis. The LR−, however, ranged from 0.17 to 0.22, and one can conclude that EP-performed PoCUS is useful to rule in appendicitis, but not sufficient on its own to rule it out. This can also be concluded from the latest three studies not included in the above-mentioned meta-analyses [138–140].

Concentric rings on ultrasound of the small bowel indicate intussusception in children in whom one suspects this condition [141]. We identified one prospective observational study and one retrospective analysis of EP-performed PoCUS for intussusception after only short periods of training, both showing high specificities of 94–97%, but varying sensitivities of 85–100% [141, 142]. One retrospective study was limited by its design giving an absence of true negative findings, but showed sensitivity of 79% in novices and 90% in a certified paediatric EP [143], while a South Korean group found that PoCUS significantly reduced the door-to-reduction time and overall stay in their ED [144].

Small bowel obstruction can be seen using ultrasound by identifying features such as small bowel dilation, abnormal peristalsis, small bowel wall oedema, and intraperitoneal free fluid [145]. Four studies in the ED showed sensitivities from 88 to 98% [145–148], with two studies showing a higher sensitivity, but lower specificity for EPs than for radiologist ultrasound when compared to CT [146, 147]. One of the studies showed lower specificity than the other three studies (54% vs 84–94%), citing a shorter didactic session and experience requirements as a possible explanation [145].

One small study found that GPs had 100% agreement with radiologists on the use of PoCUS for finding ascites on indication [93].

A small study (*n* = 50) compared ultrasound measured transverse diameter of the rectum against Roma III criteria for constipation in children, and found high sensitivity of 86%, but a somewhat low specificity of 71% [149]. However, ultrasound was not less sensitive than abdominal X-ray (87%) and trended towards being more specific (71% vs 40%). A rectal diameter of 3.8 cm or greater correlated well with constipation.

Two studies were identified using several of the above-mentioned techniques to help diagnose patients presenting with abdominal pain and found an overall improvement in diagnostic accuracy compared to work-up as usual [150, 151].

### Obstetric ultrasound

Inexperienced Danish GPs had 28 of 30 measurements of gestational age (GA) within 3 days of the obstetrician performed estimate, while the final 2 were within 7 days [93]. Johansen et al. [152] found that GP’s measurements of GA in an 11 year period (*n* = 356) showed the same agreement with actual date of birth as did those of the local obstetric service (*n* = 14,550). The same agreement was found in six other GP studies between 1985 and 2001 [153–158].

Also EP measured crown-rump length (CRL), used in first trimester estimation of GA, showed in two studies correlation coefficients of 0.95–0.98 when compared with obstetric ultrasound [159, 160]. Another study found that EPs were accurate stratifying GA into before and after 24 weeks, and thus foetal potential viability if one decides to go ahead with an emergent caesarean section in patients unable to give an accurate history due to lowered consciousness [161].

One meta-analysis assessed EPs’ accuracy in diagnosing ectopic pregnancy by PoCUS, defining a positive finding as an empty uterus in a patient with a confirmed pregnancy [162]. Using this “safe” definition, the pooled sensitivity was high at 99.3%, while the specificity ranged from 42 to 89%, pooled specificity estimate not being possible to calculate due to study heterogeneity.

Another meta-analysis included six studies aimed to show whether EP-performed pelvic ultrasound on women with symptomatic early pregnancy in the ED caused a reduction in the length of stay (LOS) in the ED, and confirmed this, with a mean reduction in LOS of 74 min (95% CI 49–99) [163].

Among those visiting ED due to bleeding in the first trimester, one study showed 42% had the expectation of getting confirmation of foetal viability by ultrasound and blood work [164]. In addition to identifying an intrauterine pregnancy, confirming foetal heart activity is decisive in diagnosing a threatened or missed abortion. We identified four studies where GPs had 100% accuracy (total *n* = 295) [93, 152, 153, 165] and one study of EPs showing a sensitivity of 89% and a specificity of 100% by use of transabdominal transducer [166]. In this study, mean GA was 9.5 weeks, and only the heart activity of the very earliest pregnancies was missed when compared to a radiologist using transvaginal transducer.

Two studies (total *n* = 387) showed that both GPs and EPs had 100% accuracy in detecting foetal position in the third trimester [152, 167].

### The eye

Studies on ocular PoCUS are listed in Table 6. Retinal detachment may be seen on ultrasound as a hyperechoic line separating from the choroid while being tethered to the optic disc. One recent meta-analysis determined the test characteristics of ocular PoCUS for this condition [168]. A sub-group analysis of five studies where the provider was an EP working in the ED found a sensitivity of 94% and a specificity of 91%. One retrospective study excluded from this meta-analysis, due to its retrospective design, showed similar numbers [169], as did two more recent prospective studies [170, 171] (see Table 6).

One study was identified estimated test accuracies for the important differential diagnoses of vitreous haemorrhage and detachment, and found high total accuracy for haemorrhage and high specificity for vitreous detachment [170]. Another study evaluated 232 patients (351 eyes) after trauma (excluding obvious globe rupture), and found high accuracy for the detection of vitreous haemorrhage, lens dislocation, globe foreign body, globe rupture, and retrobulbar haematoma [171]. The same group also found high accuracy for the detection of traumatic lens dislocation in a different study 5 years previously [172].

### Soft tissue

Linear, high-frequency ultrasound can give detailed images of structures in the soft tissue, and findings from studies are summarized in Table 7. A 2017 meta-analysis included eight studies on adult and paediatric ED populations determining the accuracy of EPs using PoCUS to detect the presence of an abscess in patients presenting with signs of skin and soft-tissue infection, and found a pooled sensitivity of 96% and a specificity of 83% [173]. The pooled sensitivity of the paediatric sub-group was slightly lower at 94%, but had the same specificity. The decision of whether to lance or not was changed in 14–56% of the cases. Pre-study teaching varied from 15 min to 1 day. A 2016 meta-analysis including six studies showed the same test accuracy [174]. Another study compared EP PoCUS and CT for abscesses head-to-head and found significantly better sensitivity for PoCUS (97% vs 77%), and similar specificity (86% vs 91% with overlapping 95% confidence intervals) [175]. In a primary care outpatient setting, it has been showed that the size of abscesses was estimated incorrectly by clinical examination in 52% of cases and ultrasound changed management in 55% of cases [176]. One study compared the test accuracy of clinical examination with and without PoCUS on finding soft-tissue abscesses [177]. They found very high accuracy and no significant difference between the groups in the population for which the EP indicated that she was clinically certain about the diagnosis (*n* = 1111). However, in the uncertain cases (*n* = 105), ultrasound changed management in a quarter, appropriately so in 85% of these. Also in a paediatric ED population, it was found that ultrasound did not change the ED treatment failure rate, even though ultrasound changed management from surgical to medical or vice versa in 25% of cases [178]. This is in contrary to another study in a paediatric population who did see a significant reduction in failure rate, with three times higher failure rates in the non-PoCUS vs PoCUS groups (14% vs 4%) [179]. The same group found similar rates in adults (*n* = 125), with 17% vs 3.7%, but the 95% confidence intervals showed 0–19.4% difference between the groups, leaving it barely statistically significant [180]. A US study showed that the ED length of stay was significantly reduced, by a mean of 73 min, when patients received EP PoCUS rather than radiology ultrasound [181]. They also found significant differences in the two groups on incision and drainage rate which was twice as high in the PoCUS group and rate of ED intravenous antibiotics, which was 60%.

Two small studies on the use of PoCUS for the detection of peritonsillar abscess [182] and dental abscess [183] showed near 100% test accuracy, but had wide confidence intervals due to small populations.

Two studies (*n* = 27 and *n* = 75) evaluated EP PoCUS diagnostic accuracy on paediatric soft-tissue neck masses and found a Cohen’s kappa coefficient when compared to the final diagnosis of 0.69 (95% CI 0.44–0.94) and 0.71 (0.60–0.83), respectively [184, 185].

One clinical study on the use of PoCUS for identification of soft-tissue foreign bodies showed that ultrasound identified two-thirds of all foreign bodies with a specificity of 97% [186]. There were no significant differences in performance characteristics of X-ray which showed sensitivity of 58% and a specificity of 90%.

### Musculoskeletal ultrasound

The retrieved studies on musculoskeletal ultrasound were on the ability to detect acute tendon trauma, joint fluid, shoulder dislocation, and bone fractures, and the test accuracy findings are summarized in Table 8.

Two studies studied the accuracy of EP-performed PoCUS on suspected ligamentous injuries in the ulnar part of the wrist and showed high specificity, but mixed sensitivity [187, 188], using magnetic resonance imaging (MRI) as the criterion standard. Two studies evaluating the same in the ankle showed high test accuracies against the same Ref. [189, 190]. A US study showed a higher specificity for ligamentous laceration on extremity penetrating trauma than clinical examination without ultrasound when compared to surgical exploration or MRI [191], and this study and an Iranian study [192] showed 94–100% sensitivity and specificity.

Two studies showed high specificity (both 98%) for paediatric hip effusions, but a somewhat reduced sensitivity of 80–85%, compared to a chart review or radiologist performed ultrasound [193, 194]. One study showed that 50% of planned joint aspirations were avoided after PoCUS of swollen joints [195].

One meta-analysis on the use of PoCUS on patients with shoulder dislocations included seven studies (*n* = 739), and showed 99.1% sensitivity and 99.8% specificity when compared to X-ray [196]. The accuracy was similar for associated fractures, but one could not determine the clinical significance due to wide confidence intervals.

A South Korean study found high accuracy for the detection of anterior and posterior cruciate ligament tears by PoCUS [197].

Finding or excluding a bony fracture could be a useful utility of ultrasound in a GP setting given a high enough accuracy, as X-ray is usually not immediately available and may require significant travelling for the patient. We identified three meta-analyses and 25 primary studies evaluating the test accuracy of EP-performed ultrasound on different fractures, all summarized in Table 8. The main finding is that there is generally a very high sensitivity and specificity for detecting the cortical disruption representing the fracture ultrasound, but less for fractures near joints.

Six diagnostic accuracy studies on the use of EP-performed PoCUS to detect paediatric skull fractures found sensitivities ranging from 77 to 100 and specificities from 85 to 100 [198–203].

Clavicular fractures were studied in three studies, all showing high accuracy [198, 204, 205], with false-negative cases being clinically non-significant green-stick fractures.

One meta-analysis of ultrasound for elbow fractures included a sub-group analysis of five studies where the operators were EPs, and showed a specificity of 95% and a sensitivity of 94% [206]. Elbow fractures can be identified on ultrasound by cortical disruption and/or posterior fat pad sign. The latter is rare in radial head subluxation without fractures according to a US study, indicating that PoCUS may be an adequate rule out test before reduction of the subluxation [207].

One meta-analysis assessed the test characteristics of ultrasound to detect paediatric forearm fractures [208] and found sensitivity and specificity of 93, and also two studies published since showed high accuracy [209, 210]. Another meta-analysis, also including studies with adults, showed even higher accuracy with a pooled sensitivity of 97% and a specificity of 95% [211], and also showed no significant accuracy differences between inexperienced and experienced physicians. A Turkish study published after this meta-analysis has shown similar test accuracy in adults [212].

Studies on metacarpal and phalangeal fractures showed sensitivities ranging from 79 to 100% and specificities from 87 to 98%, with the poorest accuracy for periarticular fractures and for the third and fourth metacarpal bones which are only available to scan from two surfaces [213–219]. The study of the distal phalanx fractures also assessed the accuracy of PoCUS to detect nail bed injuries before lifting the nail and visually inspecting, and found a 93% sensitivity and 100% specificity for this [218].

One study aimed to determine the combined accuracy for any tibia or fibula fracture, and found 100% sensitivity and 93% specificity against X-ray, and also found that all false positives were true positives when compared to CT, indicating a higher accuracy than X-ray [220].

One study showed poor sensitivity for navicular bone fracture [221].

One meta-analysis from 2017 [208] and two more recent studies [222, 223] all showed high accuracy in detection of fractures in the ankle malleoli. Three studies determined the accuracy of PoCUS specifically for fifth metatarsal fracture, and found total accuracies in the 90s [221, 224, 225].

## Discussion

### Strengths and limitations

This review is based on a search strategy that was designed to be comprehensive and sensitive enough to identify all relevant meta-analyses and primary research papers available, and included studies written in English, Spanish, Norwegian, and Swedish. In addition, reference lists of included studies were manually searched to identify further studies to include. However, the search only included searches through PubMed/MEDLINE, not EMBASE or similar proprietary databases. The main screening was only performed by one of the authors, which could be a source of bias.

One comprehensive systematic review only including clinical studies on the training and use of PoCUS by GPs already exists [12]. Given the scarcity of data, it was difficult to draw conclusions other than PoCUS has a potential of being a valuable tool for the general practitioner. A strength of our review is the wealth of data on GP relevant indications which we draw on from our EP colleagues. However, this may be one of the main weaknesses as well, as even though there is a considerable overlap in knowledge and skill bases, generalist approach, and even populations, there are also considerable differences. GPs tend to work more independently with less possibility of daily peer interaction, and have a broader scope of practice, not only including working with patients with conditions which require immediate action. In areas where patients can self-refer to emergency departments staffed by EPs, the pre-test probability of any given diagnosis will be different, with a skew towards more life-threatening conditions in EDs compared to those presenting to primary care run services. However, in other regions, where GPs may, indeed, be the first responder to any emergency, this may not be the case.

Nevertheless, much of a GP’s evidence-based practice, is, and will likely always be, based on work done in other fields. In fact, there are most likely relevant studies on the use of ultrasound done by, e.g., physiotherapist, sports medicine physicians, paediatricians, internal medicine specialist, surgeons, etc., which also could be relevant for GPs.

The studies identified were heterogenous and ranged from small pilot studies, through prospective and retrospective convenience sample observational studies, some randomised control trials and on to large, rigorous meta-analyses. In terms of operators, they include in some cases one expert GP or EP sonographer, while, in other cases, the operators were many, of different levels of experience, including novices, all only receiving short, specific didactic interventions. There were no attempts at formally assessing the quality of the primary studies by available quality assessment tools, but most of the meta-analyses will have had such assessment done by their respective authors.

Being a very heterogenous group of physicians, it is hard to establish an absolute list of possible indications for which any given GP may find PoCUS of clinical relevance. We think that we have created an overview where most GPs can find some areas of interest, but also acknowledge that others may criticise the exclusion of indications listed in Appendix 1.

## Conclusions

This systematic review shows that ultrasound, at the point of care, is increasingly being utilised by GPs and EPs across the world. It also shows that generalists can, given a certain level of pre-test probability, safely use ultrasound in a wide range of clinical settings to aid diagnosis. For many conditions, the sensitivity is high and can help the physician rule out a condition, while for others, the specificity is high, helping to rule in a diagnosis. For some conditions, the total test accuracy is high, and it may, in fact, be a valuable screening tool. For some conditions, such as identifying foreign bodies and in shoulder dislocations, PoCUS seems to have similar accuracy as X-ray, while for others, such as rib fractures, tibia and fibula fractures, pneumothorax, pneumonia, and in patients presenting with pleuritic pain of any cause, it seems to outperform conventional X-ray. PoCUS has also shown to decrease the length of time to diagnosis and discharge in some settings, decrease failure rates of treatment, and to aid in difficult intravenous access.

GPs are by no means a homogenous group of physicians, neither are EPs. It is likely that if many EPs can learn to safely use clinical ultrasound, so can many interested GPs, as both groups are trained and used to applying a wide range of methods to assess a wide range of patients and conditions. It is likely that the patient population will vary from GP to GP as well, as we all work in different regions with populations of different disease prevalence profiles and health service seeking behaviors. It is important for both GPs and EPs to be aware of one’s population’s characteristics and pre-test probabilities for any given condition with regards to all aspects of clinical work, including history taking, examination, and diagnostic studies. Given the varying prevalence in each clinician’s population, we, therefore, encourage the use of the likelihood ratios using Fagan’s nomogram [226], which as a pre-requisite for usage only requires an estimate of pre-test likelihood rather than having the exact same prevalence as in the respective studies from which the data were obtained.

This systematic review will potentially be a valuable reference for physicians searching for evidence for the use of PoCUS in their given primary care setting. Even though most of the studies involved ultrasound performed by EPs, we believe what has been found is relevant also in a GP setting, and is, to date, the best evidence available. We hope also that our review can be of value in showing the need for further research in a primary care setting, and we see a need for more rigorous study designs, with more studies with multi-centre, randomised and controlled designs.

## Data Availability

The data sets generated during and/or analyzed during the current study are available from the corresponding author on reasonable request.

## Appendix 1

See Table 9.

**Table 9:** Indications excluded due to less relevance for general practice

Organ	Indication
Heart	Echocardiography during resuscitation Paediatric echocardiography
Lungs	Thoracic aortic aneurysm Pulmonary embolism
Vessels	Type A dissection of the ascending aorta Ruptured abdominal aortic aneurysm
Abdomen and pelvis	Hepatic abscess Tubo-ovarian abscess Pneumonperitoneum Mesenteric ischemia
Central nervous system	Ventriculoperitoneal shunt malfunction Intracranial pressure through optic nerve sheath diametre Transcranial ultrasound for MCA perfusion
Trauma	Focused abdominal sonography in trauma (FAST)/extended FAST (eFAST) Hemothorax Pelvic fracture
Procedures	Regional nerve blocks Closed reduction of fractures under sedation Intubation Pericardiocentesis Neonatal intracranial bleeding Lumbar puncture Nasogastric tube placement verification Cystostomy
Others	Undifferentiated hypotension/dehydration Studies from prehospital emergency medical services Mass casualty trauma triage Gastric content

## Appendix 2

### Ultrasound and general practice (MeSH terms)

“ultrasonography”[MeSH Terms] AND (“primary health care”[MeSH Terms] OR “general practice”[MeSH Terms] OR “general practitioners”[MeSH Terms] OR “physicians, primary care”[MeSH Terms] OR “physicians, family”[MeSH Terms])

### Ultrasound and emergency medicine (MeSH terms)

“ultrasonography”[MeSH Terms] AND (“emergency medical services”[MeSH Terms] OR “emergency treatment”[MeSH Terms] OR “emergency medicine”[MeSH Terms] OR “emergencies”[MeSH Terms])

### Ultrasound and general practice (keywords)*

((((((ultrasonography) OR pocus) OR ultrasound)) OR echocardiography)) AND ((((((primary care physician)) OR (family practice)) OR (primary health care)) OR (family physician)) OR ((general practice) OR general practitioner))

automatically expanded by PubMed to

((((“diagnostic imaging”[Subheading] OR (“diagnostic”[All Fields] AND “imaging”[All Fields]) OR “diagnostic imaging”[All Fields] OR “ultrasonography”[All Fields] OR “ultrasonography”[MeSH Terms]) OR pocus[All Fields]) OR (“diagnostic imaging”[Subheading] OR (“diagnostic”[All Fields] AND “imaging”[All Fields]) OR “diagnostic imaging”[All Fields] OR “ultrasound”[All Fields] OR “ultrasonography”[MeSH Terms] OR “ultrasonography”[All Fields] OR “ultrasound”[All Fields] OR “ultrasonics”[MeSH Terms] OR “ultrasonics”[All Fields])) OR (“echocardiography”[MeSH Terms] OR “echocardiography”[All Fields])) AND (((((“physicians, primary care”[MeSH Terms] OR (“physicians”[All Fields] AND “primary”[All Fields] AND “care”[All Fields]) OR “primary care physicians”[All Fields] OR (“primary”[All Fields] AND “care”[All Fields] AND “physician”[All Fields]) OR “primary care physician”[All Fields]) OR (“family practice”[MeSH Terms] OR (“family”[All Fields] AND “practice”[All Fields]) OR “family practice”[All Fields])) OR (“primary health care”[MeSH Terms] OR (“primary”[All Fields] AND “health”[All Fields] AND “care”[All Fields]) OR “primary health care”[All Fields])) OR (“physicians, family”[MeSH Terms] OR (“physicians”[All Fields] AND “family”[All Fields]) OR “family physicians”[All Fields] OR (“family”[All Fields] AND “physician”[All Fields]) OR “family physician”[All Fields])) OR ((“general practice”[MeSH Terms] OR (“general”[All Fields] AND “practice”[All Fields]) OR “general practice”[All Fields]) OR (“general practitioners”[MeSH Terms] OR (“general”[All Fields] AND “practitioners”[All Fields]) OR “general practitioners”[All Fields] OR (“general”[All Fields] AND “practitioner”[All Fields]) OR “general practitioner”[All Fields])))

### Ultrasound and emergency medicine (keywords)*

((((((emergency medical services) OR emergency medicine) OR emergency treatment) OR emergency physician) OR prehospital medicine)) AND (((ultrasound)) OR (((ultrasonography) OR pocus) OR echocardiography))

automatically expanded by PubMed to

(((((“emergency medical services”[MeSH Terms] OR (“emergency”[All Fields] AND “medical”[All Fields] AND “services”[All Fields]) OR “emergency medical services”[All Fields]) OR (“emergency medicine”[MeSH Terms] OR (“emergency”[All Fields] AND “medicine”[All Fields]) OR “emergency medicine”[All Fields])) OR (“emergency treatment”[MeSH Terms] OR (“emergency”[All Fields] AND “treatment”[All Fields]) OR “emergency treatment”[All Fields])) OR ((“emergencies”[MeSH Terms] OR “emergencies”[All Fields] OR “emergency”[All Fields]) AND (“physicians”[MeSH Terms] OR “physicians”[All Fields] OR “physician”[All Fields]))) OR (prehospital[All Fields] AND (“medicine”[MeSH Terms] OR “medicine”[All Fields]))) AND ((“diagnostic imaging”[Subheading] OR (“diagnostic”[All Fields] AND “imaging”[All Fields]) OR “diagnostic imaging”[All Fields] OR “ultrasound”[All Fields] OR “ultrasonography”[MeSH Terms] OR “ultrasonography”[All Fields] OR “ultrasound”[All Fields] OR “ultrasonics”[MeSH Terms] OR “ultrasonics”[All Fields]) OR (((“diagnostic imaging”[Subheading] OR (“diagnostic”[All Fields] AND “imaging”[All Fields]) OR “diagnostic imaging”[All Fields] OR “ultrasonography”[All Fields] OR “ultrasonography”[MeSH Terms]) OR pocus[All Fields]) OR (“echocardiography”[MeSH Terms] OR “echocardiography”[All Fields])))

* To exclude indexed articles (which presumably were found by searching with MeSH terms) the keyword searches was done with the following filter:

((publisher[sb] NOT pubstatusnihms NOT pubstatuspmcsd NOT pmcbook) OR inprocess[sb] OR pubmednotmedline[sb] OR ((pubstatusnihms OR pubstatuspmcsd) AND publisher[sb])) OR pubmednotmedline[sb]

((publisher[sb] NOT pubstatusnihms NOT pubstatuspmcsd NOT pmcbook) OR pubmednotmedline[sb] OR ((pubstatusnihms OR pubstatuspmcsd) AND publisher[sb]))

inprocess[sb]

## Abbreviations

AAA: abdominal aortic aneurysm
ADHF: acute decompensated heart failure
CAD: coronary artery disease
COPD: chronic obstructive pulmonary disease
CRL: crown-rump length
CT: computed tomography
CXR: chest X-ray
DVT: deep vein thrombosis
ED: emergency department
EP: emergency physician
GA: gestational age
GP: general practitioner/family physician
LOS: length of stay
LR+: positive likelihood ratio
LR−: negative likelihood ratio
LUS: lung ultrasound
LVEF: left-ventricular ejection fraction
LVH: left-ventricular hypertrophy
MAPSE: mitral annular plane systolic excursion
MeSH: medical subject headings
MRI: magnetic resonance imaging
OOH: out-of-hours
PoCUS: point-of-care ultrasound
pro-BNP: pro-brain natriuretic peptide
PVC: peripheral venous catheter
